# Patient Preferences and Functional Outcomes of Forearm Crutches vs Hands-Free Single Crutches After Foot and Ankle Surgery: A Randomized Crossover Trial

**DOI:** 10.1177/24730114251363494

**Published:** 2025-08-23

**Authors:** Vincent Georg Werner, Christian Plaass, Sarah Ettinger, Leif Claassen, Anna Altemeier-Sasse, Lars-Rene Tuecking, Kiriakos Daniilidis, Daiwei Yao

**Affiliations:** 1 Department of Orthopedic and Trauma Surgery, DIAKOVERE Friederikenstift, Hannover, Germany; 2 Orthopedic Clinic, Hannover Medical School in DIAKOVERE Annastift, Hannover, Germany; 3 University Hospital for Orthopedics, Pius Hospital Oldenburg, Germany; 4 Orthoprofis, Hannover, Germany; 5 OTC Regensburg, Germany; 6 Fachärzte Rhein-Main, Seligenstadt, Germany

**Keywords:** midfoot surgery, hands-free crutch, forearm crutches, postoperative care

## Abstract

**Background:**

Assistive devices facilitate daily activities and recovery, and are essential for nonweightbearing ambulation after orthopaedic foot or ankle surgery.

This study aimed to compare the usability of forearm crutches (FCs) and hands-free single crutches (HFSCs) during the early postoperative recovery phase in terms of their effects on mobility, speed, safety, range, endurance, personal preference, and quality of life.

**Methods:**

This prospective randomized crossover study included 35 participants. Assessments included the 36-Item Short-Form Survey, Short Musculoskeletal Function Assessment Questionnaire, and European Foot and Ankle Society Score administered preoperatively and at 2 follow-up examinations. Patients were assigned to either device (FC or HFSC) for the first 3 weeks after surgery. The primary outcome was number of stumble events (SEs) during standardized mobility tests. For secondary outcomes (including mobility, speed, and range), patients completed clinical tests such as the 6-minute walk test, stair-climbing test, 10-m walk test, and indoor and outdoor parkour activities. Following the clinical tests, the patients provided qualitative feedback, including personal preference and overall device usage. After switching the devices, the tests were repeated at 6 weeks postoperatively.

**Results:**

Although FCs performed better in most mobility tests, patients favored HFSCs because of enhanced comfort and lower perceived exertion. Despite the physical advantages of FCs, patients tended to prefer HFSCs owing to their ergonomic benefits. The quality of life and physical function scores for both devices declined after surgery, reflecting a typical postoperative recovery phase. Younger and male patients generally performed better with HFSCs, whereas female, older, and overweight patients faced more challenges.

**Conclusion:**

FCs outperform HFSCs with respect to mobility, but patients prefer HFSCs due to comfort and reduced exertion although the clinical significance of these perceived differences remains uncertain. This underscores the need for personalized device recommendations to improve postoperative outcomes. This study highlights the complexity of device selection based on individual patient needs and preferences.

**Level of Evidence:**

Level II, prospective, randomized comparative study.

## Graphical abstract

**Graphical Abstract:**
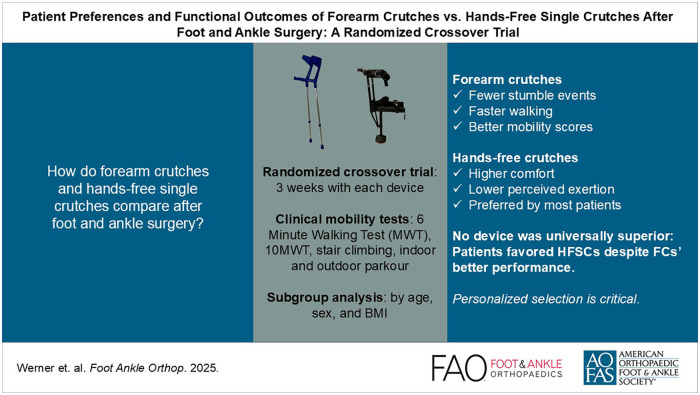

## Introduction

**Figure 1.**
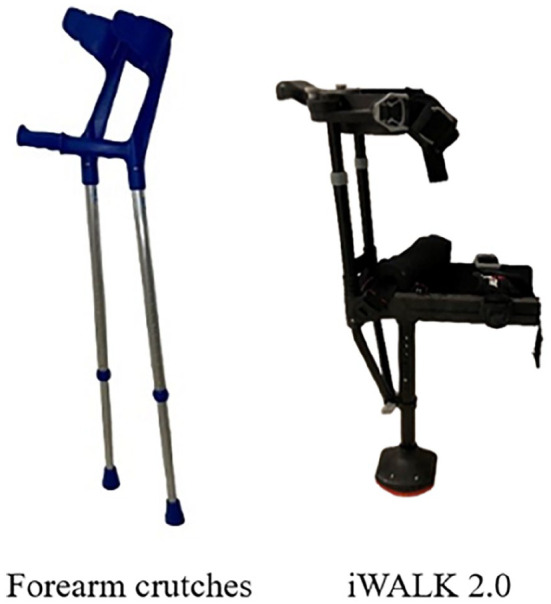
Standard forearm crutches and the hands-free single crutch (HFSC) iWalk 2.0.

**Figure 2.**
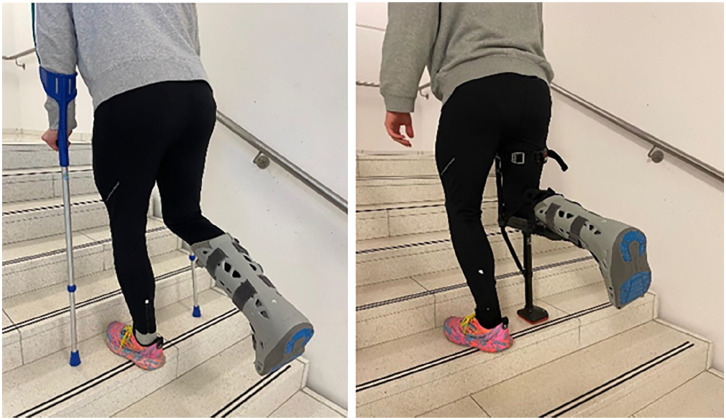
Patient using forearm crutches (left) and the hands-free single cruch (HFSC) during stair climing. In both examples the right foot is fully unloaded.

Achieving adequate nonweightbearing after foot surgery is essential.^1^ Among various available orthoses and aids, a combination of immobilization boots and forearm crutches (FCs) is usually used to achieve postoperative nonweightbearing (Figure 1). Unloading does not pose major problems in patients with adequate independent mobilization and sensorimotor coordination, and adequate compliance can be expected.^8,9^ Nonetheless, these patients may experience difficulty in managing longer walking distances owing to pain and fatigue in their upper extremities, and such difficulty may lead to reduced social contact and self-care. Patients with coordination deficits and poor general physical conditions struggle to attain adequate unloading.^20^ Patients with concomitant upper-extremity pathologies, such as rheumatoid arthritis or upper-extremity fractures, cannot unload with FCs and are often bound to wheelchairs. Consequently, several novel orthoses have been developed to overcome these limitations. The hands-free single crutch (HFSC) (iWALK 2.0; iWALKFree Inc, Long Beach, CA) allows for complete unloading of the leg distal to the knee joint by transferring the load completely to the femur (Figures 1 and 2). Although numerous studies have directly compared FCs with HFSCs, comparative data during the postoperative recovery period remain limited. Therefore, the current study aimed to compare FCs and HFSCs during the early postoperative recovery phase in terms of their effects on mobility, speed, range, endurance, safety, handling, quality of life (QoL), preference, impairment, and perceived exertion.

## Methods

This noninvasive, prospective, randomized crossover study was conducted on 35 patients who underwent midfoot or hindfoot surgery requiring (partial) unloading of the foot for at least 6 weeks.^16^ This study was approved by the ethics committee (approval no. 8542_MPG_23b_2019). Orthopaedic doctors selected the patients. All participants provided written informed consent.

The inclusion criteria were as follows: patients older than 18 years but younger than 70 years who could safely perform single-leg standing and showed no Trendelenburg sign. The exclusion criteria were as follows: balance disorders, body mass index (BMI) >35, osteoporosis, previous upper- or lower-extremity surgery prohibiting the use of FCs, substance abuse or dementia, cardiopulmonary disease, and coordinative or neuromuscular disorders that could limit mobilization.

**Figure 3.**
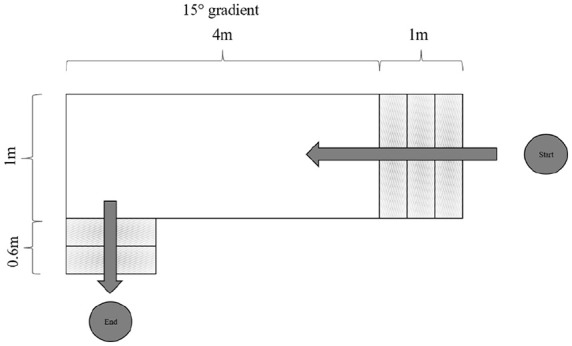
The InP activity consisted of a 3-step ascent and descent and a track with a slight incline of approximately 4 m in length.

Before study inclusion, candidates completed an indoor parkour (InP) activity using their HFSCs. The InP activity consisted of a 3-step ascent and a track with a slight incline of approximately 4 m in length (Figure 3). The InP activity was performed only after a sufficient level of safety had been demonstrated based on a previously established rating on a numeric rating scale (NRS) ranging from 1 to 10. The NRS score must be at least 5/10 for safety, as applied in our pilot study.^3,23^

Patients were randomly divided into 2 groups: group A (n = 17) used HFSCs for the first 3 weeks and FCs for the final 3 weeks, whereas group B (n = 18) used FCs for the first 3 weeks and HFSCs for the final 3 weeks. A block-randomized list (block sizes 4-8) created using the R statistical software and the blockrand package was used to perform randomization and assign patients to their respective treatment groups.

**Figure 4.**
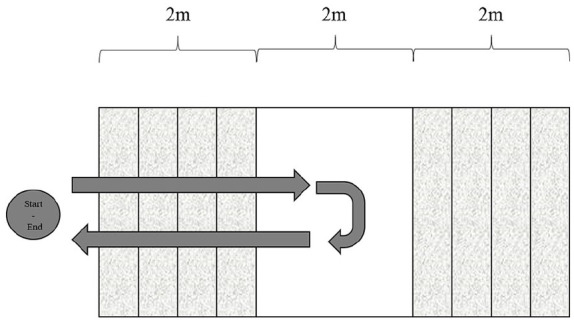
The two stair-climbing test consisted of a five step ascent and an 5 step descent.

**Figure 5.**
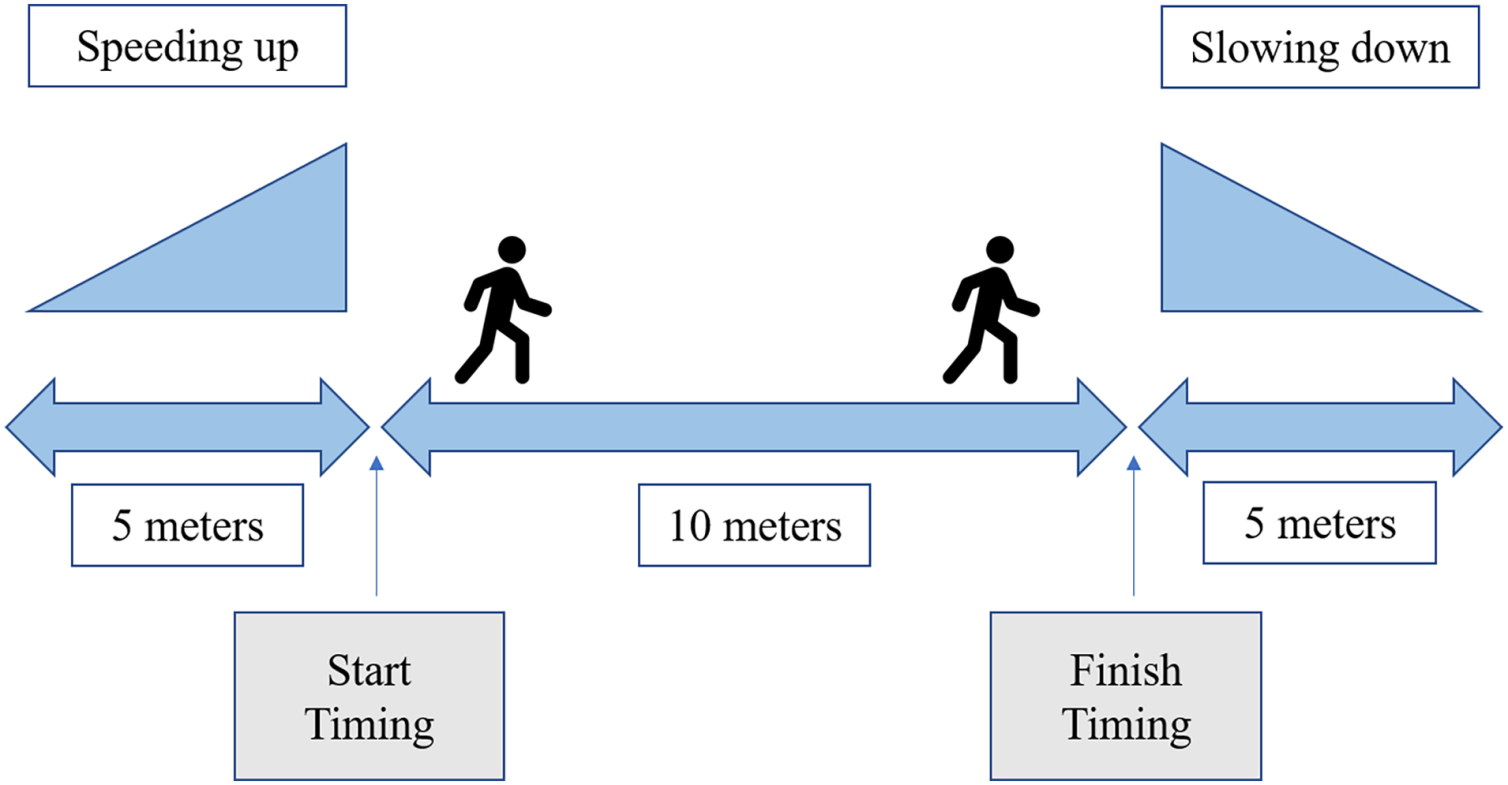
In the 10-meter walk test, participants start with a 5-meter acceleration phase, followed by a 10-meter walking distance over which the time is measured.

**Figure 6.**
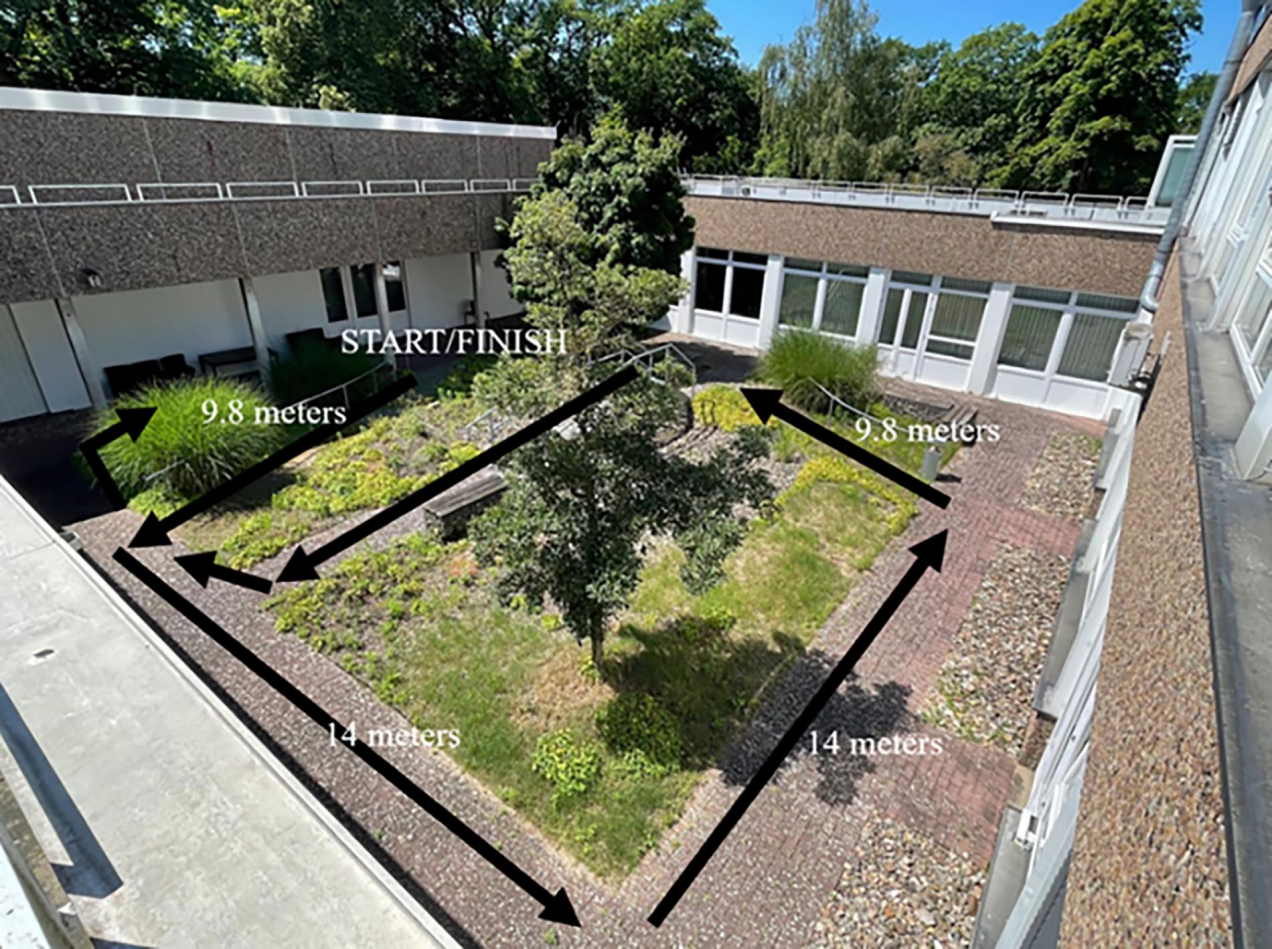
The outdoor course used in the study comprised various pavement types and included ascents, descents, and stairways.

**Figure 7.**
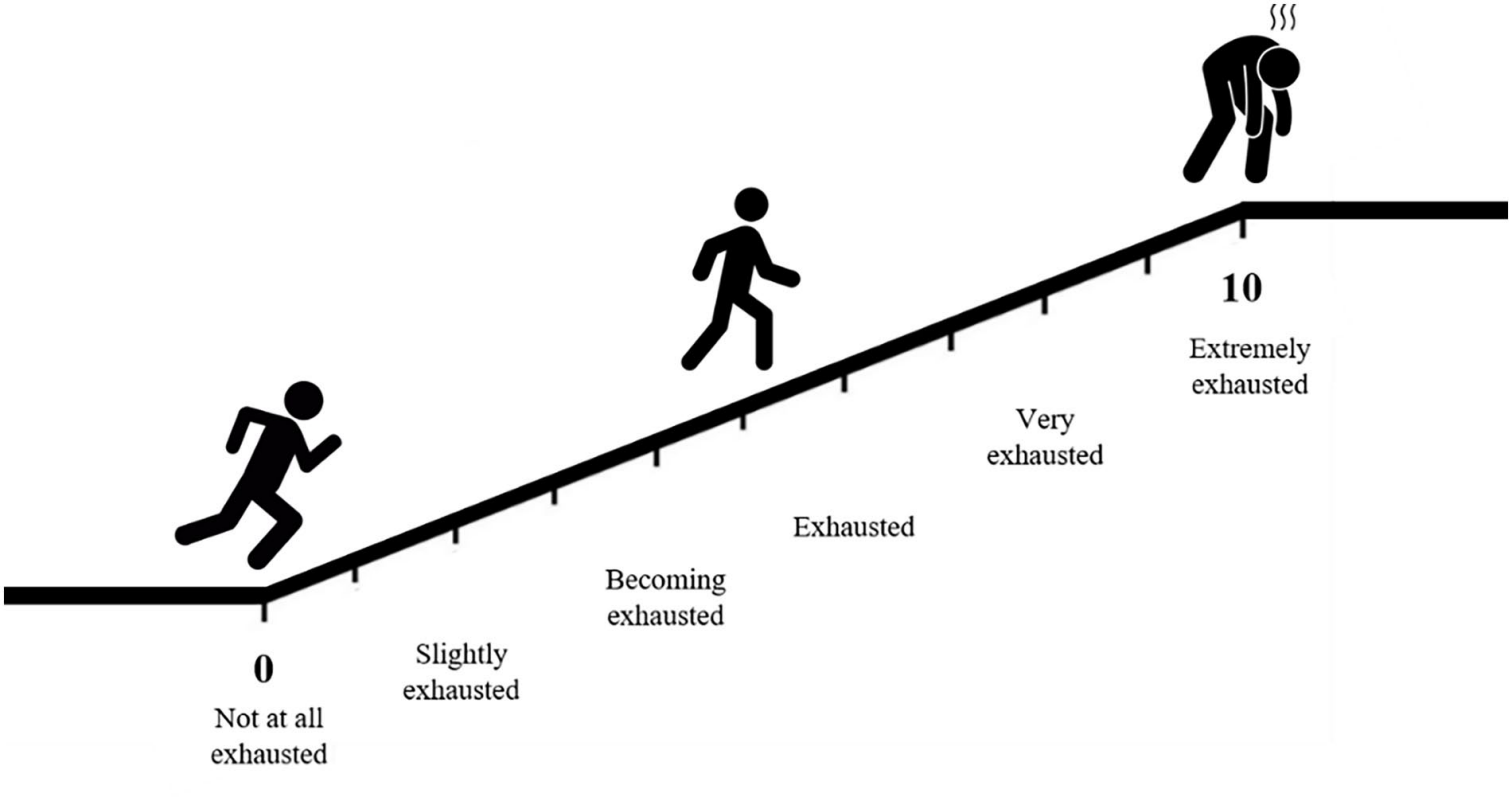
The perceived exertion was assessed using the Overall Measure Numeric Intensity (OMNI) scale.

**Figure 8.**
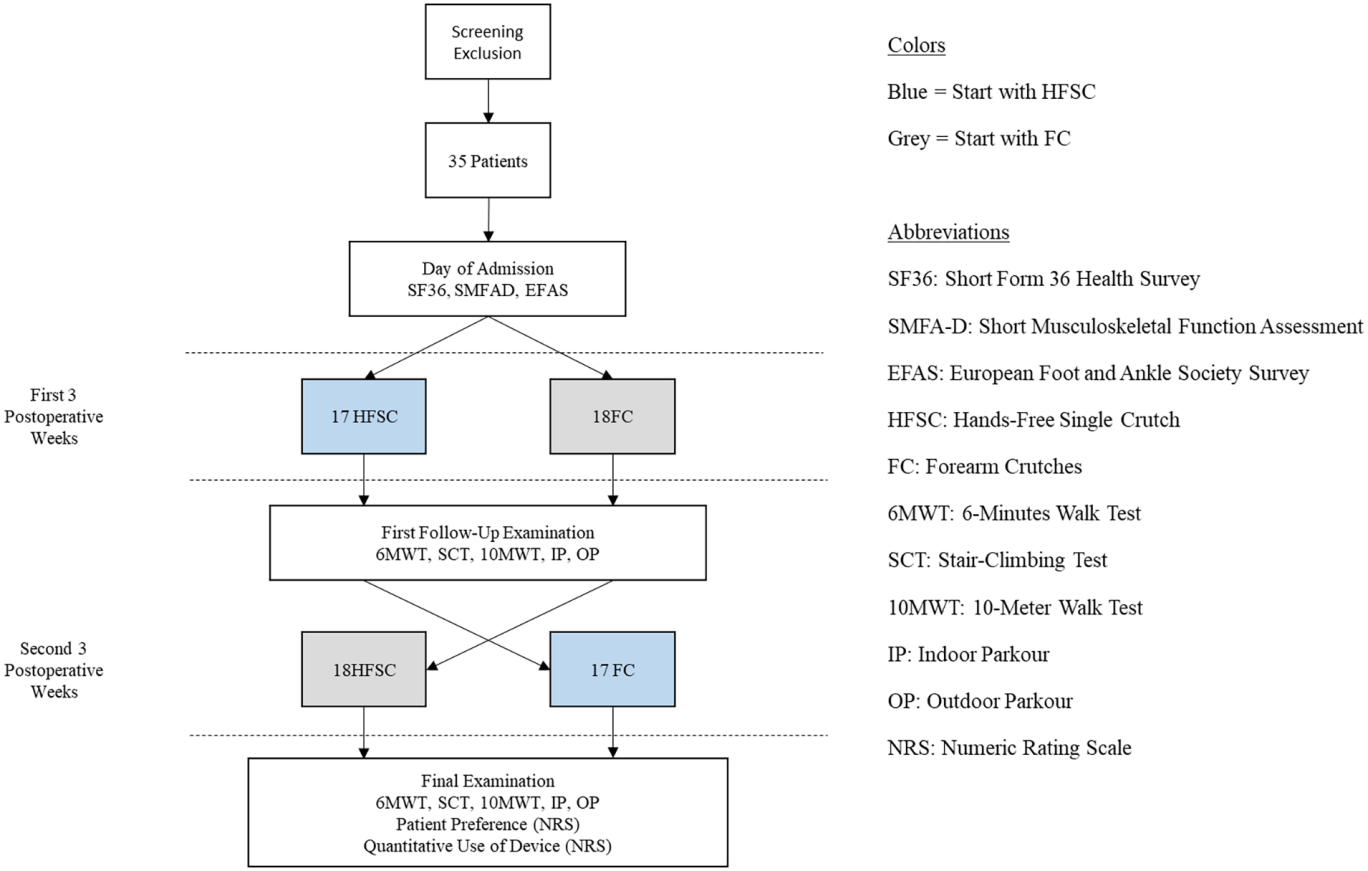
Study flowchart illustrating patient enrollment, allocation to hands-free single crutch (HFSC) or forearm crutches (FC), and follow-up assessments including 6MWT, SCT, 10MWT, indoor and outdoor parcours, and patient-reported outcomes.

Patients were assessed both preoperatively and at 3 and 6 weeks postoperatively using the 36-Item Short Form Health Survey (SF-36),^10^ Short Musculoskeletal Function Assessment Questionnaire (SMFA-D),^18^ and European Foot and Ankle Society Score (EFAS).^15^ At 3 weeks, the patients undertook the 6-minute walk test (6MWT) on a flat paved road,^2^ stair-climbing test (Figure 4), 10-m walk test (10MWT; Figure 5),^14^ outdoor parkour (OuP) activity (Figure 6), and InP activity. The OuP activity included a stair-tread element, a section with sand, a gravel path section, and a paved section. The primary outcome measure was the number of stumble events (SEs) observed during mobility tests. In these tests, stumble events (SEs) were defined as “a clearly visible interruption in the gate process for more than 1 second.”^23^ This endpoint was pre-specified to capture real-world stability and safety in postoperative ambulation. Additional measures included total time to complete the task (in seconds), distance (in meters), perceived exertion (Overall Measure Numeric Intensity [OMNI] scale), and patient-reported feedback. Immediately after completing the test, the patients rated how exhausted they were after using each device on an OMNI exertion scale, ranging from 0 (“not at all exhausted”) to 10 (“extremely exhausted”; Figure 7).^19^ Sufficient time for full recovery was allowed between the tests. At the end of the first follow-up examination, the patients rated their levels of comfort, personal recommendations, safety, pain, and all-day use of the respective orthosis on a visual analog scale (VAS).^3^ The entire testing procedure was repeated at the second follow-up examination. Before final discharge, the patients were asked for their subjective feedback and overall preferences for HFSC, FC, neither, or both (Figure 8).

### Statistical Analysis

A previous case number calculation performed on the number of SEs indicated 1.1 SEs (SD, 1.2) when participants used HFSCs, compared with 1.9 SEs (SD, 1.2) when they used FCs; the correlation between these SE values was 0.085. According to Cohen *d*, the effect size was 0.50. A 2-sided paired *t* test revealed that the minimum group size was 35 patients for a significance level of 5% and test strength of 80%. The evaluation for the current study used SE frequency with a *t* test at an alpha significance level of .05 (ie, 5%).

The mean values of all variables were calculated with descriptive statistics. In addition to a comparison between HFSCs and FCs in the entire cohort, the cohort was divided into subgroups according to sex (male vs female), age (≤36 years vs >36 years [where the median age was 36 years]), and weight (BMI ≤25 vs >25 [where BMI >25 indicated overweight]). Additionally, differences were analyzed between subgroups using a *t* test, equal variance was checked using the Levene test, and normal distribution was examined using the Smirnov goodness-of-fit test. All tests were performed using SPSS software version 28.0.1.1 (IBM Corp, Armonk, NY). In the case of incomplete data sets (eg, patient dropouts for the 6-week postoperative period), mean value imputation (mean values of orthosis groups at respective time points) was applied, and the sensitivity of the analysis to these assumed data points was checked.

Additionally, a sensitivity analysis using multiple imputation was performed for stumble events to assess the robustness of the results. Because of the small sample size and the proportion of missing data, this approach led to highly variable and thus limited interpretable results. Therefore, the primary analysis was based on mean value imputation.

## Results

The cohort comprised 15 male and 20 female patients, with a mean age of 39 (20-63) years, a mean BMI of 26.96 (20.28-34.72), mean weight of 80.49 (55-115) kg, and a mean height of 1.72 (1.55-1.92) m.

The number of SEs (the primary outcome) was significantly lower when patients used FCs compared to HFSCs (0.65 vs 0.24, *P* < .007). This suggests a superior performance of FCs in terms of gait stability. In addition, patients walked further during the 6MWT using FCs (376.85 vs 313.81 m, *P* < .000) but reported a lower level of perceived exertion according to the OMNI scale (OMNI scale scores: 4.85 vs 5.98, *P* < .011; Table 2).

Patients completed the InP activity significantly faster with FCs than with HFSCs in the preoperative clinical testing (15.51 vs 19.85 s, *P* < .0001) and after 3 weeks of use (11.75 vs 15.09 s, *P* < .000). The speed in the InP activity increased compared with preoperative testing, with notable improvements for both HFSC use (19.85 vs 15.09 s, *P* < .000) and FC use (15.51 vs 11.75 s, *P* < .000). The number of HFSC-related SEs decreased significantly after 3 weeks of use compared with that before surgery (0.07 vs 0.26, *P* < .042).

Patients were significantly faster in the 10MWT when using FCs than when using HFSCs (3.95 vs 5.24 s, *P* < .000). They also completed the OuP activity faster with FCs than with HFSCs (35.53 vs 46.40 s, *P* < .000; Table 1). Direct comparison of FCs and HFSCs in the InP activity indicated no significant differences.

**Table 1.** Overview of Clinical Test Results: HFSC vs FC.

	FC (n = 35)	HFSC (n = 35)	*P* Value
Indoor parkour: time, s	11.75	15.09	<.001
SE	0.03	0.07	.484
Outdoor parkour: time, s	35.53	46.40	<.001
SE	0.21	0.10	.208
10MWT: time, s	3.95	5.24	<.001
6MWT: distance, m	376.85	313.81	<.001
SE	0.24	0.65	.007
Stair-climbing test: time, s	17.37	16.69	.568

Abbreviations: FC, forearm crutch; HFSC, hands-free single crutch; 6MWT, 6-minute walk test; 10MWT, 10-m walk test.

The SF-36 scores for both devices decreased postoperatively; however, those for HFSCs were significantly higher than those for FCs (50.89 vs 45.39, *P* < .032; Table 2). Moreover, the SMFA-D function scores were higher (worse) for both HFSCs (39.01 vs 19.08, *P* < .000) and FCs (41.44 vs 19.08, *P* < .000) compared to preoperative testing. The SMFA-D disability scores were also higher (worse) for both HFSCs (40.63 vs 22.74, *P* < .000) and FCs (47.67 vs 22.74, *P* < .000) postoperatively.

**Table 2.** Overview of Health Questionnaire Results: HFSC vs FC.

	FC (n = 35)	HFSC (n = 35)	
Perceived exertion in the 6MWT	5.98	4.85	.011
SF-36	45.39	50.89	.032
EFAS	9.345	10.37	.293
SMFA-D			
Functional index	41.44	39.01	.255
Bother index	47.67	40.63	.069

Abbreviations: EFAS, European Foot and Ankle Society Score; FC, forearm crutch; HFSC, hands-free single crutch; SF-36, 36-Item Short Form Health Survey; 6MWT, 6-minute walk test; SMFA-D, Short Musculoskeletal Function Assessment Questionnaire.

The overall all-day usage of FCs was higher than that of HFSCs (VAS 0-10, 8.935 vs 5.715; *P* < .000; Table 3). Patients expressed higher comfort levels with HFSCs than with FCs (6.782 vs 4.875, *P* < .000), and they were more likely to recommend HFSCs than FCs (7.932 vs 6.516, *P* < .006). Further analysis was conducted by dividing the study population according to sex (male vs female), age (≤36 vs >36 years), and BMI (BMI ≤25 vs >25).

**Table 3.** Test Results for the VAS (1-10) Comparing Comfort, Safety, Pain, Personal Recommendation, and Usage: HFSC vs FC.

Independent Samples *t* Test	FC (n = 35)	HFSC (n = 35)	*P* Value
Comfort	4.88	6.78	<.001
Pain	3.65	4.05	.534
Safety	6.37	6.7	.384
Personal recommendation	6.52	7.93	.006
All-day usage	8.94	5.72	<.001
Personal preference, %	44.8	51.7	

Abbreviations: FC, forearm crutch; HFSC, hands-free single crutch; VAS, visual analog scale.

In the 10MWT, men were significantly faster than women with HFSCs (4.66 vs 5.73 s, *P* < .033; Table 4). The time difference between the two (time HFSC – time FC) was significantly greater in women (1.71 vs 0.73 s; *P* < .0179). Men were significantly faster than women with HFSCs in the OuP activity (40.93 vs 51.51 s, *P* < .028), and the time difference in completing the course (time HFSC – time FC) was also smaller (7.17 vs 16.21 s; *P* < .006).

**Table 4.** Overview of Clinical Test Results With Personal Characteristics According to Sex and With Score Differences Between Male and Female.

	Sex		
Test	Male	Female	*P* Value
6MWT			
OMNI FC	4.80	4.77	.968
OMNI HFSC – OMNI FC	−1.65	−0.93	.403
10MWT			
Time HFSC, s	4.66	5.73	.033
Time HFSC – time FC, s	0.73	1.71	.018
OuP			
Time HFSC, s	40.93	51.51	.028
Time HFSC – time FC, s	4.37	16.21	.006
InP			
Time HFSC, s	13.95	16.31	.097
Time HFSC – time FC, s	3.43	3.53	.948
Frequency of HFSC usage	5.70	5.64	.945

Abbreviations: FC, forearm crutch; HFSC, hands-free single crutch; InP, indoor parkour; OMNI, Overall Measure Numeric Intensity; OuP, outdoor parkour; 6MWT, 6-minute walk test; 10MWT, 10-minute walk test; VAS, visual analog scale.

Women scored significantly higher (worse) on the SMFA-D functional index (41.87 vs 35.24, *P* < .047) and impairment index (45.05 vs 35.07, *P* < .029) in 3 weeks after surgery. The frequency of FC use (score range: 1-10) was significantly higher in women than in men (9.25 vs 7.17, *P* < .051). Younger patients completed the 10MWT significantly faster than older patients with HFSCs (4.56 vs 5.97 s, *P* < .004) and FCs (3.49 vs 4.44 s, *P* < .008; Table 5); they were also significantly faster than older patients when using the HFSCs in the InP activity (13.17 vs 17.13 s, *P* < .005) and OuP activity (40.55 vs 52.60 s, *P* < .014).

**Table 5.** Overview of Clinical Test Results with Personal Characteristics According to Age and with Score Differences Between Those Aged ≤36 Years and Those Aged >36 Years.

	Age		
Test	≤36 y	>36 y	*P* Value
6MWT			
OMNI FC	7.05	4.85	.003
OMNI HFSC – OMNI FC	−2.30	0.11	.003
10MWT			
Time HFSC, s	4.56	5.97	.004
Time HFSC – time FC, s	1.07	1.53	.276
OuP			
Time HFSC, s	40.55	52.60	.013
Time HFSC – time FC, s	8.16	13.91	.197
InP			
Time HFSC, s	10.89	12.67	.145
Time HFSC – time FC, s	2.28	4.46	.136
Frequency of HFSC usage	5.25	6.20	.325

Abbreviations: FC, forearm crutch; HFSC, hands-free single crutch; InP, indoor parkour; OMNI, Overall Measure Numeric Intensity; OuP, outdoor parkour; 6MWT, 6-minute walk test; 10MWT, 10-minute walk test; VAS, visual analog scale.

In the OuP activity, older patients stumbled significantly more often when using FCs (0.35 vs 0.10, *P* < .038). In the 6MWT, younger patients covered longer distances when using HFSCs (343.00 vs 282.90 m, *P* < .021) but were subjectively more exhausted according to the OMNI scale when using FCs (7.05 vs 4.85, *P* < .003). When categorized by weight, lighter patients achieved significantly better results (Table 6).

**Table 6.** Overview of Clinical Test Results With Personal Characteristics According to Weight and With Score Differences Between BMI ≤25 and BMI >25.

	Weight (BMI)		
Test	BMI ≤25	BMI >25	*P* Value
6MWT			
OMNI FC	5.35	6.27	.265
OMNI HFSC – OMNI FC	0.29	−1.78	.019
10MWT			
Time HFSC, s	5.27	5.23	.942
Time HFSC – time FC, s	1.34	1.27	.888
OuP			
Time HFSC, s	45.62	46.76	.597
Time HFSC – time FC, s	11.46	10.72	.880
InP			
Time HFSC, s	14.58	15.32	.645
Time HFSC – time FC, s	2.46	3.74	.419
Frequency of HFSC usage	6.65	5.29	.189

Abbreviations: BMI, body mass index; FC, forearm crutch; HFSC, hands-free single crutch; InP, indoor parkour; OMNI, Overall Measure Numeric Intensity; OuP, outdoor parkour; 6MWT, 6-minute walk test; 10MWT, 10-minute walk test; VAS, visual analog scale.

## Discussion

The present study evaluated the outcomes between HFSCs and FCs in patients recovering from midfoot or hindfoot surgery.

The primary outcome (number of SEs) was significantly lower with FCs, indicating greater stability in dynamic mobility tasks. This key finding suggests a potential stability advantage conferred by FCs despite other subjective benefits offered by HFSCs. As our pilot study^23^ already suggested, our results demonstrated that FCs outperform HFSCs in most mobility tests (reflected in the higher speed, fewer SEs, and greater distance traveled per time), including the 6MWT, 10MWT, and InP and OuP activities. Despite the superior performance of FCs in clinical tests, patients favored HFSCs for comfort even though the average difference in OMNI perceived exertion scores (1.13 points) may not be clinically meaningful, and most patients had more experience with FCs. This familiarity may have conferred a performance advantage to FCs, while HFSCs, despite less efficient test metrics, were viewed more favorably by patients—likely because of perceived ergonomic benefits and reduced upper extremity strain during daily use.

The superior performance of FCs could be attributed to patient reliance on a “swing-through gait,” which allows for a longer stride.^7,17^ This enabled the patients to complete the tests in less time. Moreover, most patients had prior experience with FCs, which may have influenced their proficiency and comfort with these devices.

Furthermore, HFSC users reported higher SF-36 scores during recovery, indicating possible benefits for perceived quality of life in the early postoperative phase. While this represents a statistically significant result, the clinical relevance remains uncertain. A 5.5-point difference is unlikely to represent a meaningful impact for patients or clinicians.

Although FC users achieved superior clinical performance, their heavier reliance on the upper extremities likely contributed to discomfort, pain, and fatigue, negatively affecting subjective assessments. Additionally, the recommendation and comfort scores for HFSC were significantly higher, suggesting that these devices may address key patient concerns, particularly during longer recovery periods. Nonetheless, QoL scores (SF-36 and SMFA-D) decreased postoperatively for both devices compared with preoperative values, likely as a result of postoperative pain and immobilization. This implies that the physiological and psychological state of the patients can differ considerably when they are healthy or have recovered from surgery.^21^

Despite covering shorter distances, the results indicated that HFSCs are more energy-efficient, as suggested by the perceived exertion scores and as previously illustrated by the lower oxygen consumption and heart rates recorded during treadmill tests at a speed of 1.5 km/h.^23^ A related study confirmed that HFSC use is associated to lower energy expenditure than unaided walking or FC use.^12^ Conversely, this could explain the higher QoL scores associated to HFSC use, a result supported by Martin et al,^11^ who noted higher preferences for HFSCs in preoperative assessments of 2 orthoses because of reduced fatigue. The design of FCs, which does not mimic natural walking patterns, leads to inefficiencies and higher energy consumption resulting from the additional loads that they place on both upper and lower extremities, activating more muscles and increasing overall energy use.^22^ HFSCs support a 2-point alternating gait pattern that facilitates energy-efficient reciprocal walking in a way that allows both lower limbs to contribute to propulsion and movement without any reliance on the upper extremities.^5,11^ At the same time, this gait modality requires users to take each step sequentially, with contact made only through the healthy foot and the foot of the orthosis, which demands greater physical balance from the user, as the challenge of coordinating movement with an assistive device depends mainly on the motion of the contralateral lower extremity.^13^ In contrast, FCs provide triple support (3-point gait),^24^ offering greater stability and quicker movement, which likely explains the lower number of SEs in the 6MWT. Despite lower perceived exertion, the average difference in the OMNI Scale of 1.13 points may not reflect a clinically meaningful change in perceived exertion. Therefore, these findings should be interpreted with caution and regarded as exploratory signals rather than definitive clinical implications.

Furthermore, our study highlights the notable differences in device adaptation among demographic groups. Men and younger participants tended to adapt more quickly to both devices, whereas women faced greater challenges with HFSCs, as reflected in their poorer outcomes on functional and impairment indices. These differences may be attributed to greater upper body strength and physical fitness in men, which facilitate smoother adaptation to HFSC.

Despite better performance across all tests, younger patients reported higher levels of exertion with FC, indicating that although FC offers superior speed, it may not be sustainable for younger, more active individuals. These findings suggest that demographic factors should be considered in orthopaedic device selection and patient counseling.^6^

### Study Limitations

Although SEs provide a relevant indicator of gait security, our method of counting SE may not have captured all clinically relevant disturbances in balance. Furthermore, the main study exclusively included patients without coordination deficits or severe mobility limitations, potentially limiting the generalizability of the findings to a broader patient population, including those with severe disabilities or significant upper-body strength limitations. The exclusion of patients with balance disorders or substantial cardiopulmonary disease restricts the applicability of our findings to clinically diverse settings.

Another potential bias is the familiarity of participants with FCs. Because most patients were already familiar with FC use owing to previous injuries, their degree of comfort and proficiency using these devices might have influenced their performance and subjective feedback, skewing the results in favor of the more familiar device. In contrast, the short adaptation period required for HFSCs may be insufficient for patients to overcome the initial learning curve associated with their use. Although patients were randomized, there was no stratification according to previous experience with the device. A stratified analysis or covariate adjustment for prior experience could have helped to isolate the true effect of each device by minimizing this bias.

Thus, our results may not reflect all the potential benefits of HFSCs that might be achieved through prolonged familiar use, which in turn would imply improved outcomes over time.

Challenges noted with the use of the HFSC in real-world environments, such as partial incompatibility with certain types of orthopaedic boots (VACOped boots; OPED GmbH, Bavaria, Germany) and issues associated with narrow living spaces, suggest limitations to the practical applicability of HFSCs. All patients had to use both orthopaedic devices in combination with the VACOped boots (VACOped + FC or VACOped + HFSC). Patients reported that the use of VACOped boots sometimes led to pain resulting from pressure on the front lower leg when placed on the HFSC plateau. This might have led to a lower overall use and less training time.

Furthermore, other than HFSC, FCs were available at all times postoperatively for patients, providing backup for HFSC. This availability allowed patients to use both devices interchangeably, potentially influencing higher QoL scores for HFSC and a lower frequency of HFSC use. During the critical 4-6-week postoperative period, when increased loading on the foot is required, the limitations of HFSCs, including the fact that they only allow for full unloading, may have led to switching to FCs in some patients. This need would help explain the prevalent use of FCs, because HFSCs are not suitable for continuous use under increased load conditions. Additionally, familiarity with FCs likely affected performance and comfort ratings. Stratified randomization or covariate adjustment for prior use could reduce this bias in future studies. Finally, the patient feedback concerning QoL is a subjective assessment, and could therefore be susceptible to individual perceptual errors and placebo effects.^4^

## Conclusion

This study found that HFSCs did not match FCs in terms of gait stability or short-term functional performance, but were generally preferred because of enhanced comfort levels and perceived quality of life, particularly by younger and more physically active patients. Although HFSCs underperformed in most clinical tests over a 3-week period, their benefits in daily life and mobility enhancement, particularly for younger, more active individuals, highlight the need to consider individual patient factors when selecting assistive devices.

These patient experiences suggest that although HFSCs can reduce fatigue and improve QoL, their practicality depends on compatibility with other medical devices as well as on the environment and demographics of the patients. Such variations in patient experiences underscore the need for ongoing research efforts to refine orthotic designs and tailor training methods in order to better accommodate diverse patient needs and improve the usability of assistive devices in postoperative care. We also emphasized the importance of aligning device choices with patient-specific characteristics to improve compliance and overall satisfaction.

## Supplemental Material

sj-pdf-1-fao-10.1177_24730114251363494 – Supplemental material for Patient Preferences and Functional Outcomes of Forearm Crutches vs Hands-Free Single Crutches After Foot and Ankle Surgery: A Randomized Crossover Trial Supplemental material, sj-pdf-1-fao-10.1177_24730114251363494 for Patient Preferences and Functional Outcomes of Forearm Crutches vs Hands-Free Single Crutches After Foot and Ankle Surgery: A Randomized Crossover Trial by Vincent Georg Werner, Christian Plaass, Sarah Ettinger, Leif Claassen, Anna Altemeier-Sasse, Lars-Rene Tuecking, Kiriakos Daniilidis and Daiwei Yao in Foot & Ankle Orthopaedics

## References

[1] American Orthopaedic Foot & Ankle Society. How to be non-weightbearing after surgery. Accessed January 20, 2025. https://www.footcaremd.org/resources/how-to-help/how-to-be-non-weightbearing-after-surgery

[2] Bennell K Dobson F Hinman R. Measures of physical performance assessments: Self-Paced Walk Test (SPWT), Stair Climb Test (SCT), Six-Minute Walk Test (6MWT), Chair Stand Test (CST), Timed Up & Go (TUG), Sock Test, Lift and Carry Test (LCT), and Car Task. Arthritis Care Res (Hoboken). 2011;63(suppl 11):S350-S370. doi:10.1002/acr.20538 22588756

[3] Chiarotto A Maxwell LJ Ostelo RW Boers M Tugwell P Terwee CB. Measurement properties of visual analogue scale, numeric rating scale, and pain severity subscale of the brief pain inventory in patients with low back pain: a systematic review. J Pain. 2019;20(3):245-263. doi:10.1016/j.jpain.2018.07.009 30099210

[4] Friesen P. Towards an account of the placebo effect: a critical evaluation alongside current evidence. Biol Philos. 2020;35(11):1-23. doi:10.1007/s10539-019-9733-8

[5] Goldberg B. Atlas of orthoses and assistive devices. Accessed July 15, 2024. https://cir.nii.ac.jp/crid/1130000798145730560

[6] Hall N Parker D Williams A. An exploratory qualitative study of health professional perspectives on clinical outcomes in UK orthotic practice. J Foot Ankle Res. 2020;13(1):49. doi:10.1186/s13047-020-00416-w 32727515 PMC7392713

[7] Heisel J. Physikalische therapie und rehabilitation. In: CJ Wirth W Mutschler D Kohn , et al, eds. Praxis der orthopädie und unfallchirurgie. Georg Thieme Verlag; 2014:119-121.

[8] Hershko E Tauber C Carmeli E. Biofeedback versus physiotherapy in patients with partial weight-bearing. Am J Orthop (Belle Mead NJ). 2008;37(5):E92-E96. 18587509

[9] Hustedt JW Blizzard DJ Baumgaertner MR Leslie MP Grauer JN. Current advances in training orthopaedic patients to comply with partial weight-bearing instructions. Yale J Biol Med. 2012;85(1):119-125. 22461750 PMC3313526

[10] Lins L Carvalho FM. SF-36 total score as a single measure of health-related quality of life: scoping review. SAGE Open Med. 2016;4:2050312116671725. doi:10.1177/2050312116671725 PMC5052926 27757230

[11] Martin KD Unangst AM Huh J Chisholm J. Patient preference and physical demand for hands-free single crutch vs standard axillary crutches in foot and ankle patients. Foot Ankle Int. 2019;40(10):1203-1208. doi:10.1177/1071100719862743 31375043

[12] Nagpurkar A Troeller A. An evaluation of crutch energetics using standard and “hands free” crutches. Accessed July 15, 2024. https://iwalk-free.com/wp-content/uploads/2015/06/Evaluation-of-crutch-energetics.pdf

[13] O’Sullivan SB. Perceived exertion. A review. Phys Ther. 1984;64(3):343-346. doi:10.1093/ptj/64.3.343 6366836

[14] Peters DM Fritz SL Krotish DE. Assessing the reliability and validity of a shorter walk test compared with the 10-Meter Walk Test for measurements of gait speed in healthy, older adults. J Geriatr Phys Ther. 2013;36(1):24-30. doi:10.1519/JPT.0b013e318248e20d 22415358

[15] Richter M Agren PH Besse JL , et al. EFAS score - multilingual development and validation of a patient-reported outcome measure (PROM) by the score committee of the European Foot and Ankle Society (EFAS). Foot Ankle Surg. 2018;24(3):185-204. doi:10.1016/j.fas.2018.05.004 29933960

[16] Schumacher M Schulgen G. Kapitel 17: cross-over-studien-design. In: M Schumacher, & G Schulgen, eds. Methodik klinischer studien. Springer; 2002:305-317.

[17] Shoup TE Fletcher LS Merrill BR. Biomechanics of crutch locomotion. J Biomech. 1974;7(1):11-19. doi:10.1016/0021-9290(74)90065-7 4595087

[18] Swiontkowski MF Engelberg R Martin DP Agel J. Short musculoskeletal function assessment questionnaire: validity, reliability, and responsiveness. J Bone Joint Surg Am. 1999;81(9):1245-1260. doi:10.2106/00004623-199909000-00006 10505521

[19] Utter AC Robertson RJ Green JM Suminski RR McAnulty SR Nieman DC. Validation of the Adult OMNI Scale of perceived exertion for walking/running exercise. Med Sci Sports Exerc. 2004;36(10):1776-1780. doi:10.1249/01.mss.0000142310.97274.94 15595300

[20] Vasarhelyi A Baumert T Fritsch C Hopfenmüller W Gradl G Mittlmeier T. Partial weight bearing after surgery for fractures of the lower extremity–is it achievable. Gait Posture. 2006;23(1):99-105. doi:10.1016/j.gaitpost.2004.12.005 16311201

[21] Vögele C Steptoe A. Physiological and subjective stress responses in surgical patients. J Psychosom Res. 1986;30(2):205-215. doi:10.1016/0022-3999(86)90051-6 3723451

[22] Waters RL Mulroy S. The energy expenditure of normal and pathologic gait. Gait Posture. 1999;9(3):207-231. doi:10.1016/s0966-6362(99)00009-0 10575082

[23] Yao D Meyer-Kobbe L Ettinger S , et al. Functional, spiroergometric, and subjective comparisons between forearm crutches and hands-free single crutches in a crossover study. Foot Ankle Orthop. 2023;8(2):24730114231172734. doi:10.1177/24730114231172734 PMC10201150 37223637

[24] Yap W Hairodin Z Kwek E. Axillary versus forearm crutches: a prospective cohort comparing which is superior for 3-point crutch gait. Malays Orthop J. 2021;15(2):36-42. doi:10.5704/MOJ.2107.006 PMC8381660 34429820

